# Chronic ischemic heart disease: A nonuniform syndrome^☆^

**DOI:** 10.1016/j.ahjo.2026.100744

**Published:** 2026-03-07

**Authors:** Samir Alam, Carl J. Pepine

**Affiliations:** a American University of Beirut Medical Center, Beirut, Lebanon; b Department of Medicine, University of Florida, Gainesville, FL, United States of America

**Keywords:** Coronary disease, Plaque biology, Coronary CT, Plaque features

## Abstract

Ischemic heart disease (IHD) is a leading cause of morbidity and mortality worldwide. Decades of progress have focused on identification of obstructive coronary plaque (“stenosis” >50–70% diameter narrowing) as a threshold for diagnosis and target for therapy. Emerging evidence underscores the significance of a myriad of relevant isolated or coexisting biologic, physiologic, and radiologic mechanisms and features, beyond stenosis, as causes of ischemia and hence predictors of ischemia-related clinical outcomes. In the context of the chronic ischemic syndrome, a persistent challenge lies in the disconnect between obstructive plagues and symptoms, clinical outcomes, and responses to revascularization and guideline-based medical therapy. For instance, a study elucidating some mechanisms observed a 2.6- fold increase in identification of a nonobstructive cause for myocardial ischemia among symptomatic patients referred for invasive coronary angiography.

This review aims at highlighting emerging impactful elements and mechanisms and proposes a broad spectrum of phenotypes, which better capture the heterogeneous characteristics of chronic coronary artery disease (CAD). We examine nontraditional factors, radiometric features, polygenic and genetic signatures, and biomechanical plaque characteristics, which underpin numerous coexisting and overlapping pathologic and clinical manifestations. The focus shifts from quantifying mechanical obstruction to identifying biologically active plaques, which may not be confined to Acute Coronary Syndromes but are present across the spectrum of chronic CAD. Recognizing these features carries important prognostic and therapeutic implications.

## 1 Introduction

Despite enormous advances in pharmacologic management and technologic interventions, ischemic heart disease (IHD) remains a leading cause of death worldwide [1]. Whereas Acute Ischemic Syndromes trigger the larger share of events, Chronic ischemic syndromes, traditionally viewed as relatively benign, encompass many diverse pathologic and biologic phenotypes but portend substantial morbidity and adverse events. As such, there are many key unanswered questions and challenging unmet needs. Principal among them are the imperfect benefits of revascularization, and persistence of symptoms following optimal conventional medical treatment [2], [3], [4]. A large body of evidence validates the impact of plaque characteristics on physiological, biologic, and clinical course and outcome. Of relevance and contrary to conventional thinking, it is becoming evident that high risk and adverse plaque characteristics are not confined to patients with Acute Coronary Syndromes but are prevalent among patients considered stable and chronic [5].

## 2 Special physiologic considerations

### 2.1 Context of ischemia matters: pathophysiologic and mechanistic considerations, ischemia and re-perfusion

Physiologic and clinical evidence indicate that inducible myocardial ischemia is a crude determinant of clinical outcome of patients with CAD, in stark contrast to primary flow-reduction-ischemia, which often leads to myocardial injury, infarction and arrhythmic death [6]. The difference can be explained, at least in part by physiological mechanisms triggered by low-flow myocardial ischemic conditions such as coronary spasm or coronary thrombosis. In this context, within minutes of flow reduction, the ATP-dependent function of cardiomyocyte Na^+^/K^+^-ATPase and re-uptake of norepinephrine into sympathetic nerve terminals are impeded. Loss of Na^+^/K^+^-ATPase function induces a local increase in extracellular K^+^, which can depolarize neighboring cardiomyocytes. Sudden reperfusion following profound myocardial ischemia further promotes ventricular arrhythmias by highly heterogeneous reperfusion on the microvascular level. Hence, identifying and preventing acute ischemic surrogates constitutes an important challenge and a critical paradigm in mitigating IHD (Table 1).

**Table 1:** Chronic coronary artery disease.

Category	Phenotype	Description
Asymptomatic	NONOBSTRUCTIVE	Non-obstructive plaques (<50% stenosis), no symptoms.
	Silent obstructive	Obstructive lesions (≥50% stenosis) without anginal symptoms.
Symptomatic	Obstructive CAD	Significant coronary obstruction causing angina or angina-equivalent symptoms.
	Mixed (obstructive + NONOBS)	Coexisting obstructive and non-obstructive plaques with high plaque burden.
	Coexisting microvascular disease (coexist-MVD)	Epicardial CAD with concurrent microvascular dysfunction causing symptoms.

### 2.2 Endothelial dysfunction and microvascular disease/dysfunction

Coronary microvascular dysfunction, highly prevalent among women and symptomatic patients. This contributes to ischemia in ≥50% of women with symptoms (ANOCA) and/or signs (INOCA) of ischemia in the absence of obstructive CAD [7]. Endothelial dysfunction leads to impaired vasodilation and increases oxidative stress, promoting thrombogenesis and adverse cardiovascular events [8]. Microvascular dysfunction is also commonly present in patients with the syndrome of angina and no obstructive CAD (ANOCA) or ischemia and no obstructive CAD (INOCA) [9]. Hence, current guidelines emphasize the need for targeted treatment in patients with (INOCA), incorporating endothelial function testing and tailored pharmacologic interventions. Therapies such as ACE inhibitors/receptor blockers, statins, calcium channel blockers and endothelin receptor blockers/antagonists are gaining prominence in managing microvascular angina [10], [11]().

### 2.3 Special populations and clinical challenges

#### 2.3.1 IHD in women

In addition to shared mechanisms and conventional clinical characteristics, several gender peculiarities exist. Post-menopausal hormonal changes exacerbate the influence of existing cardiovascular risks. Furthermore, Clonal Hematopoiesis of Indeterminate Potential, CHIP, a condition of mutations of blood progenitor cells that can potentially increase the risk of inflammation, cardiovascular disease, cancer, and mortality. Premature natural menopause is linked to a higher prevalence of CHIP [12].

#### 2.3.2 Cancer and IHD

CAD and cancer share common mechanisms and pathogenic factors. Furthermore, cancer treatments, including chemotherapy, targeted therapy and radiation, affect endothelial function, potentiate atherosclerosis and can have deleterious effects on atherosclerosis, bleeding and thrombosis [13].

#### 2.3.3 Sudden death in the young population

Subclinical atherosclerosis in young individuals often remains undetected until sudden cardiac events occur. Understanding and identifying individuals at-risk through elaborate screening is crucial, particularly in those with a strong family history of premature CAD or sudden cardiac death. Genetic testing, focused imaging studies and early lifestyle interventions may be beneficial in these populations [14], [15].

#### 2.3.4 Genetic and environmental influences

Conventional, polygenic risk factors and social determinants, including lifestyle, diet, physical inactivity contribute to individual susceptibility to IHD. The role of epigenetics in modifying risk profiles is also an important area of ongoing research.

#### 2.3.5 Psychosocial and socioeconomic determinants

Factors such as chronic stress, social isolation, and economic instability have been identified as important contributors to cardiovascular disease risk. Addressing these factors through targeted interventions may improve long-term cardiovascular outcomes [16].

#### 2.3.6 Inflammation and atherosclerosis

Chronic inflammation plays a pivotal role in plaque formation, destabilization and acute ischemic events. Biomarkers such as high-sensitivity CRP and interleukins serve as important markers and mediators of risk. Systemic inflammatory diseases, among them rheumatoid arthritis and psoriasis, have been linked to accelerated atherosclerosis and biologic destabilization. In a study of patients with recent NSTEMI, a high baseline hs-CRP levels were associated with the presence of pan-coronary atherosclerosis and focal high-risk plaques [17], [18].

#### 2.3.7 Biologic, physiologic, and radiometric targets beyond coronary stenosis

Gould et al. demonstrated that a focal coronary artery narrowing of approximately 50% (by quantitative analysis) reduces maximal coronary blood flow, leading to ischemia under conditions of increased myocardial oxygen demand. Vessel lumen narrowing >50% (or > 70% by visual estimation) have been deemed “significant,” and clinical studies have traditionally implemented these angiographic thresholds to define the presence and severity of coronary heart disease (CHD) [19]. Adopting these thresholds in the management of CAD, large-scale clinical trials have shown that in patients with stable coronary artery disease (CAD), optimal medical therapy is noninferior to contemporary revascularization strategies, including percutaneous coronary intervention (PCI) with modern stents and surgical techniques. PCI of angiographically determined lesion stenosis of >70%, has not consistently demonstrated a survival benefit in stable CAD, except in patients with high-risk coronary anatomy.

Fractional flow reserve (FFR), a physiological index incorporating anatomical, microvascular, and hemodynamic factors, offers a more integrative assessment of coronary flow and pressure gradients across stenoses, thereby improving decision-making beyond angiographic severity alone. CCTA defined plaque characteristics correlate with functionally significant lesions. Notwithstanding however, FFR-guided PCI demonstrated reduction of ischemia driven events without impacting survival [20], [21].

### 2.4 Interaction of coronary plaque imaging and physiology

Plaque composition significantly affects physiologic parameters such as FFR and CFR and interacts with clinical outcomes. Plaque characteristics, derived by coronary CT angiography (CCTA) or intravascular imaging (e.g., OCT or IVUS): Low attenuation plaque (<30 HU), positive remodeling, Napkin-ring sign, spotty calcifications, large lipid core, thin-cap fibroatheroma (TCFA), and microchannels or neovascularization were demonstrated to significantly impact the clinical outcome of patients with CAD beyond severity of stenosis [22], [23].

Integrating high-risk plaque (HRP) features and Fractional Flow Reserve (FFR) was demonstrated to influence the lesion-oriented outcome of FFR-based assessment. In a study by of 697 vessels from 458 patients, (81% with stable CAD), non-ischemic lesions, qualitative (ql-HRP) and quantitative (qn-HRP) showed a synergistic impact on risk assessment and had prognostic interactions with FFR and treatment modalities [22]. Integrating plaque features and physiologic assessment can have added impact on risk stratification and the optimization of a treatment strategy. Whereas FFR evaluates physiologic significance, HRP features assess morphologic vulnerability. Integrating the two elements leads to better risk stratification and tailored treatment [23].

## 3 Clinical implications

FFR measurements should not be interpreted as strictly dichotomous values. Instead, they should be considered within a comprehensive assessment that encompasses anatomical characteristics, plaque morphology, and patient-specific biological factors. This contextualized approach enables more clinically meaningful and personalized decision-making.

### 3.1 Challenge of NOBSTRUCTIVE CAD

Atherosclerotic disease may be non-obstructive and clinically silent. Coronary narrowing of <50% (diameter reduction) has been considered “not significant” or “nonobstructive” CAD. New insights into mechanisms leading to ACS underpin the notion that acute coronary events are commonly not caused by slow, progressive arterial lumen narrowing, but rather by sudden blood flow obstruction due to plaque disruption (plaque erosion or rupture) and associated vascular thrombosis; the culprit lesions are most often nonobstructive in severity [17]. In the PROMISE (Prospective Multicenter Imaging Study for Evaluation of Chest Pain) trial, the majority (77%) of cardiovascular deaths and myocardial infarctions occurred in patients with <50% lumen stenosis at baseline. Similarly, the ICONIC (Incident Coronary Syndromes Identified by Computed Tomography) study found that most acute coronary syndromes arise from nonobstructive lesions. This data suggests that by focusing on patients with “significant” or “obstructive” disease, many patients escape opportunity to address of appropriate preventive treatment [17], [24]. Not surprisingly, focusing on treating coronary stenoses using coronary angioplasty or stenting has not resulted in lower mortality or reduced risk of myocardial infarction in patients with stable CHD. The recently concluded 10 year follow up of SCOTT-Heart revealed improved outcome utilizing CCTA, attributed to superior identification of non-obstructive plaque and hence effective preventive treatment and better-targeted medical therapy rather than to increased revascularization. Additionally, microvascular and endothelial dysfunction underpin a significant proportion of patient with obstructive and nonobstructive disease. Utilization of novel diagnostic protocols incorporating multimodal imaging and invasive testing is mandatory for better clinical outcomes [25].

### 3.2 CCTA and intra-vascular imaging

CCTA is rapidly evolving to become a cornerstone in diagnosis and management of stable CAD. With advancing technology, expanding clinical utility, guideline endorsements and the advent of AI facilitated plaque characterization and functional assessment integration, CCTA has become integral to cardiovascular care and clinical decision-making. Plaque characterization and identification of high- risk plaques (HRP) detect features that place lesions and patients at risk for future adverse events [26]. The SCOT-HEART demonstrated that CCTA- HRP detection impacted adverse coronary events with ∼40% reduction in coronary heart disease death or nonfatal MI (primary endpoint) with no overall increase in revascularization at 5 years [23]. At 10 years, there was sustained CV Benefit - a 21% relative reduction in death/MI attributed to better preventive and medical treatment without increasing overall revascularization rates [27], [28].

CCTA findings and relevance in identifying high-risk plaque features was compared to OCT. Defining HRP as a plaque with at least 2 of the following 4 features: positive remodeling (PR), low-attenuation plaque (LAP), Napkin-ring sign (NRS), and Spotty calcification (SC), The presence of high-risk plaque (HRP) on coronary computed tomography angiography (CCTA) closely mirrored findings on optical coherence tomography (OCT) and was predictive of future cardiac events, highlighting its clinical utility in risk stratification [5], [29]. Similarly, the prognostic Implications of Angiographically Derived Coronary Radial Wall Strain in Diabetic Patients and Non–Flow-Limiting Stenosis was evaluated in comparison to optical coherence tomography-detected vulnerability features (OCT-VFs); a post hoc analysis of the COMBINE OCT-FFR dataset. The primary endpoint was lesion-oriented composite endpoint (LOCE), a composite of cardiac death, target vessel-related myocardial infarction, and clinically driven target lesion revascularization. In diabetic patients with non–flow-limiting stenosis, RWS can help to localize stenoses with OCT-VFs. RWS predicts increased risk for LOCE, both independently from and incrementally beyond OCT-VFs [28], [30].

### 3.3 Future perspectives

Current evidence, the prevention and the clinical assessment of CAD must encompass a comprehensive list of relevant elements that include integration of genetic, biomarkers, clinical, physiological, imaging aspects.AI-driven algorithms allows for enhanced interpretation of imaging studies, identifying high-risk patients, and predicting cardiovascular events with greater accuracy than traditional methods [31], [32].

### 3.4 Evolving therapeutic strategies

The approach to managing CAD must evolve, from lesion focused to patient centered, and from emphasis on revascularization to early detection prevention and intervention. In addition to above-described advances in diagnosis and plaque characterization, important pharmacological advancements must be incorporated including highly effective and validated existing treatment, novel therapies targeting inflammation (e.g., siRNA, PCSK9 inhibitors, colchicine) shown to be effective in reducing major adverse cardiovascular events. Personalized treatment approaches, integrating genomic and proteomic data, are enhancing treatment precision. While PCI and CABG remain mainstays for symptomatic obstructive CAD, non-obstructive disease and symptoms mandate a comprehensive preventive and therapeutic algorithm addressing underlying biologic, radiometric and physiologic targets. SGLT2I and GLP1-A [33].

## 4 Conclusions

Chronic Coronary Artery Disease (CAD) is a heterogeneous syndrome with varied mechanisms and clinical presentations. Therefore, a shift in approach is necessary—one that incorporates comprehensive diagnostic and therapeutic strategies integrating anatomic, biologic, physiological, and imaging (radiometric) data. This integrative model is expected to improve both the prevention and treatment of IHD. In line with these considerations, we propose a novel classification system for Chronic Ischemic Disease that reflects its multifactorial characteristics and supports more precise, personalized clinical decision-making.

## CRediT authorship contribution statement

**Samir Alam:** Writing – review & editing, Writing – original draft, Conceptualization. **Carl J. Pepine:** Writing – review & editing.

## Ethics statement

No IRB approval needed.

## Declaration of competing interest

All authors have no conflict of interest.
